# Round the Hospitals

**Published:** 1921-01-22

**Authors:** 


### ROUND THE HOSPITALS.

After nearly twenty-five years' service as matron of the Radcliffe Infirmary, Oxford, Miss A. J. Watt is retiring. She has done great service for the Infirmary, where many changes, developments, and improvements have been made during the last quarter of a century, including extensions to the buildings in 1906 and the building of a new wing which was opened in 1913. The number of beds has increased during Miss Watt's matronship from 139 to 170, and the present high average number occupied, 169, is a measure of the activity and efficiency which pervades the whole institution. During the war sixty-five extra beds were provided for soldiers, and Miss Watt was Principal Matron of the 3rd Southern General Hospital, receiving from His Majesty the Royal Red Cross. The matronship, which is advertised in our columns this week, carries with it a salary of £220, which, be it noted, is £80 less than the minimum recommended by the College of Nursing for hospitals of its size.

No institution is keener in attracting voluntary help and interest than King's College Hospital. A movement has lately been inaugurated at East Dulwich with the object of naming and maintaining a bed at this institution, and Mrs. Drayson, whose successful efforts on behalf of the Alexandra Guild and the Treloar Hospital at Alton have given her wide experience, has inaugurated a women's association with a strong committee for this purpose. A series of entertainments is to be given with the object of immediately raising £200. They opened the campaign agreeably with a New Year party for children at St. Barnabas Hall, Dulwich, when Mrs. Drayson explained to the company the aims of the new society, and they have now followed it up with a fancy-dress ball. It is a scheme which might well be followed in other localities. Dancing for money is certainly more exhilarating than begging for it.

The annual prize-giving at the Training-school of the Royal Southern Hospital, Liverpool, took place on the 11th inst., and was an unusually festive occasion. It marked the opening of the new extension to the Nurses' Home by the President, Dr. Thomas Woodsend, who announced on this occasion two munificent gifts from anonymous donors. The first consisted of Victory Bonds to the value of £10,000; the donor intimated that he favoured the

**ROUND THE HOSPITALS**—(*continued*).

social and educational benefit of the nurses. The use of the income from this capital sum for the future of the training-school will therefore benefit largely from this noble gift. The second gift of *x*-ray apparatus was a token of gratitude for services rendered by a member of the honorary staff to members of the donor's family. Miss Bagnall, the matron, entertained a large company to tea, after which the nursing staff carried out a capital programme of songs, dances, and sketches previously given for the amusement of the patients at Christmas. Mrs. Woodsend then presented the prizes. The Gold Medal for five years' service was won by Miss M. S. Freeborn, who was the former Silver Medallist. Miss B. J. J. Tracey carried off the Silver Medal, and Miss J. F. Wall the Matron's Book Prize for first-year nurses. The new accommodation for the nurses enables the hours on duty to be shortened and the conditions generally much improved. It opens up a new era of prosperity for the school.

The advent of Miss Cardwell, recently appointed matron at the Cumberland Infirmary, Carlisle, was marked by a double reception on the last days of the year. The first night the guests were the friends of the nurses, the committee, and the staff, when a concert, at which many of the patients were present, was given by members of the day and night staffs. On New Year's Night Miss Cardwell's guests included the Mayor of Carlisle, the Dean and Mrs. Rashdall, and many other well-known supporters of the hospital. Dr. Barnes, President, who was unable to be present, wrote a letter marked by real understanding of nursing ideals in which he announced that, with the cordial concurrence of the Matron, he proposed to give each year a gold medal to the best all-round nurse at the close of her third-year's training, consideration being given to conduct, discipline, efficiency, and economy. Dr. Lidyard, who presided in the absence of Dr. Barnes, spoke very warmly of Miss Cardwell, whom he welcomed to the Infirmary; she had been treasured in the place she came from and it was up to them in Carlisle to treasure her in the same way.

The South African Trained Nurses' Association has now become an incorporated body, a privilege which gives it the right to exercise all the functions of an individual or duly registered company, either in courts of law, or before Parliament, or in connection with other corporate bodies. The immediate control of busines is in the hands of the Central Governing Board, which is representative of all the Branches belonging to the Association. As in this country with the College of Nursing, difficulty arises in regard to making representation a reality. But the great distances in South Africa between the Branches render the danger of over-centralising more insistent. The S.A.T.N.A. has accomplished so much for nursing in the time since it was started that we feel confident these difficulties will be happily surmounted. We advise members to hold up criticism for a year and give the Association time to get used to its new status.

A new South Indian Nursing Association has been formed in Madras, with Lady Willingdon, O.B.E., as President, and forms part of a scheme for the development of nursing and the founding of nursing homes in that area. From April next Government hospitals in Madras city will be closed to all but the poor, or those officially entitled to medical treatment by Government medical officials. The new Nursing Association will be affiliated to Lady Ampthill's nurses' institution, and a large increase in the number of nurses is in contemplation to meet all demands.

The Saint Barnabas Guild for Trained Nurses is in great need of funds for developing its admirable work, and Lady Henry Somerset is promoting a bazaar in aid, which is to be held on February 7 and 8, at 46 Upper Grosvenor Street, W., kindly lent for the occasion by Mrs. Hartog. Princess Marie Louise has promised to attend on the first day and open the bazaar. The Duchess of Rutland, the Marchioness of Londonderry, Countess Beauchamp, and other well-known people are among the patronesses, and it promises to be a great success.

A verdict of manslaughter was returned against Thomas Edward Wilcock, a male nurse from a Manchester institution, at the conclusion of the inquest, held on January 6, on Canon Banham, aged eighty-eight. Wilcock had had charge of the deceased Canon, and evidence was given to show that on the night preceding the death he came on duty in an excited state, and was heard to threaten his patient. Dr. Fryer gave evidence that the cause of death was exhaustion resulting from injuries to the mouth. He could conceive of no accident which could have caused the whole of the injuries.

The British Red Cross Library, 48 Queen's Gardens, Lancaster Gate, W. 2, is appealing for children's books for the use of sick children in hospitals all over Great Britain. At this season a bewildering number of annuals and gift books descend upon the happy few, and they are entreated to overhaul their book-shelves and send their surplus store to their less fortunate brothers and sisters. Disused "Readers" would be very suitable, too, for this purpose.

Marylebone Infirmary has a very musical reputation, and every time the musical instruments have to be tuned someone is sure to make a protest, for, alas! all these little domestic details have to be settled in public. The rumour has started that there are some two dozen harmoniums in the institution, and Miss Broadbent, Chairman of the Infirmary Committee, has had to explain there are but thirteen all told and two small organs, which of course need periodical tuning and adjustment. Those who carp at the number should go and see the infirmary, which is in the nature of a small city. If nothing cost more than harmony in the Poor-law budget, rates would be appreciably lower.

